# An IMU-Aided Body-Shadowing Error Compensation Method for Indoor Bluetooth Positioning

**DOI:** 10.3390/s18010304

**Published:** 2018-01-20

**Authors:** Zhongliang Deng, Xiao Fu, Hanhua Wang

**Affiliations:** School of Electronic Engineering, Beijing University of Posts and Telecommunications, Beijing 100876, China; dengzhl@bupt.edu.cn (Z.D.); whh0710@bupt.edu.cn (H.W.)

**Keywords:** Bluetooth indoor positioning, body-shadowing detection, error compensation model, range-based positioning

## Abstract

Research on indoor positioning technologies has recently become a hotspot because of the huge social and economic potential of indoor location-based services (ILBS). Wireless positioning signals have a considerable attenuation in received signal strength (RSS) when transmitting through human bodies, which would cause significant ranging and positioning errors in RSS-based systems. This paper mainly focuses on the body-shadowing impairment of RSS-based ranging and positioning, and derives a mathematical expression of the relation between the body-shadowing effect and the positioning error. In addition, an inertial measurement unit-aided (IMU-aided) body-shadowing detection strategy is designed, and an error compensation model is established to mitigate the effect of body-shadowing. A Bluetooth positioning algorithm with body-shadowing error compensation (BP-BEC) is then proposed to improve both the positioning accuracy and the robustness in indoor body-shadowing environments. Experiments are conducted in two indoor test beds, and the performance of both the BP-BEC algorithm and the algorithms without body-shadowing error compensation (named no-BEC) is evaluated. The results show that the BP-BEC outperforms the no-BEC by about 60.1% and 73.6% in terms of positioning accuracy and robustness, respectively. Moreover, the execution time of the BP-BEC algorithm is also evaluated, and results show that the convergence speed of the proposed algorithm has an insignificant effect on real-time localization.

## 1. Introduction

The indoor location-based service (ILBS) has recently gained considerable attention due to its social and commercial values, and its market value is predicted to be worth $10 billion by 2020 [1]. Meanwhile, the demands for accurate localization in indoor environments have increased dramatically [2,3]. Various positioning technologies, including Infrared [4], Ultrasonic [5], Ultra-Wideband (UWB) [6], Pseudolite [7], Wireless Local Area Network (WLAN) [8], and Bluetooth Low Energy (BLE) [9], have been proposed, aiming to improve the localization performance indoors. BLE positioning has aroused great research attention recently because of its stable signal with low fluctuation, easy implementation with low energy cost, low computational complexity, and high mobility [9]. Meanwhile, an ABI research report on Bluetooth has shown that Bluetooth devices will break 5 billion shipments by 2021 [10], promoting BLE positioning to one of the most promising solutions for indoor accurate and robust localization.

BLE indoor positioning can be divided into two different schemes: fingerprint-based and range-based. Both schemes rely on the Received Signal Strength (RSS) of the Bluetooth signals. The fingerprint-based scheme consists of two stages: the off-line stage and the on-line stage. In the off-line stage, the RSS from the anchor points (APs) at test points are collected (also named fingerprints) and a fingerprint database is then constructed. In the on-line stage, matching algorithms, such as the Weighted K-Nearest Neighbor (WKNN) and the Bayesian-based matching, are implemented to estimate the unknown points’ (UPs) coordinates on the basis of the measured RSS and fingerprint database. Although fingerprint-based BLE positioning could achieve relatively high positioning accuracy, one meter in proximity mode [11], the collection and construction of the database would not be energy-efficient and environment-adaptive. The range-based positioning scheme calculates the distances between APs and a UP on the basis of the measured RSS and the Path-Loss Model (PLM), which can be called RSS-based ranging, and conducts trilateration to obtain the coordinates of the UP. The RSS-based ranging is lightweight and more flexible than fingerprinting, and the trilateration method could achieve more accurate positioning results when the PLM is correctly modified [12]. However, the RSS easily fluctuates because of the obstacles present indoors, which are, especially, the human bodies.

Bluetooth signals are predominantly transmitted in the 2.4 GHz frequency band, which is also the resonance frequency of water [13]. The human body is made of about 72% water [14], therefore the Bluetooth signals are significantly absorbed when transmitting through human bodies. This absorption causes a large decay in the RSS of Bluetooth signals, which, as a result, would bring considerable errors in RSS-based position estimations. Reports show that most ILBS users spend 70%–90% of their time in indoor or urban areas [15], which indicates that the body-shadowing impairment is an unavoidable problem when implementing indoor BLE positioning.

The effect of the human bodies on fingerprint-based positioning has been studied in [16,17,18]. Researchers in [16] identified the effects of user body’s facing on the RSS as a source of errors in location estimation, and their experimental results indicated that the RSS at a given location varies about 5 dBm depending on the direction of the user facing. The works in [17] added another source of errors for the RSS measurements related to the effect of the human body, especially the hands, considering that the mobile devices are held by the users. Fet et al. [18] analyzed the signal attenuation by the human body in fingerprinting-based positioning systems, and proposed a signal attenuation model which is able to generate the fingerprints for multiple orientations. All these works have great significance on fingerprint database construction and on the implementation of fingerprint-based positioning. However, these works would not be adaptable enough in range-based positioning systems. Researches on the impairment of range-based positioning systems due to body-shadowing have also been done in [19,20,21]. Della Rosa et al. [19] analyzed the effect of the human body, and especially the hand, on RSS distance measurements, and concluded that a model for body-shadowing should depend on several factors, such as the dimensions of the body and the composition of human tissues. The works in [20] regarded body-shadowing according to different channel models which have different channel parameters, and gave the parameters of the body-shadowing channels through empirical data and a manual differentiation of the channels. The researchers in [21] studied the human-induced effects on RSS ranging measurements under different shadowing states, and showed the human body effects on RSS-based ranging and position estimation through experiments. Although these works have given a comprehensive analysis on the effects of body-shadowing on range-based positioning, they either needed a manual work to a detect shadowing situation, or did not provide an effective method to mitigate the body-shadowing error when implementing range-based BLE positioning. Researches considering the human body effect in other domains can also been found in [22,23,24]. Kilic et al. [22] analyzed the effect of a human body based on time-of-arrival (TOA) measurements conducted in static UWB experiments in the 3–5.5 GHz band. Schmitt et al. [23] analyzed the effect of body-shadowing on Radio Frequency-based (RF-based) localization results and focused on incident management. Cotton et al. [24] proposed a shadowed fading model that is capable of characterizing shadowed fading in wireless communication channels for device-to-device communications.

Since previous works have not given an effective method to detect body-shadowing situations and to mitigate the shadowing error, our work aims to propose a body-shadowing error compensation method with shadowing autonomous detection for indoor range-based BLE positioning and to improve both the positioning accuracy and the robustness of the system. This paper firstly analyzes the body-shadowing influence on RSS-based ranging and derives a mathematical expression of the relation between the body-shadowing effect and the positioning error. Then, a body-shadowing situation detection strategy based on the heading information obtained from Inertial Measurement Units (IMU) is designed and evaluated. Meanwhile, an error compensation model together with a novel Bluetooth positioning algorithm with body-shadowing error compensation (BP-BEC) is established and proposed in this paper to mitigate the positioning error. The experiments were conducted in indoor body-shadowing environments, and the results demonstrated the improvements of the proposed method. The remainder of this paper is arranged as follows: Section 2 describes the system model of BLE positioning and analyzes the body-shadowing influence on both RSS-based ranging and positioning; Section 3 designs the body-shadowing detection strategy, establishes the error compensation model, and studies the BP-BEC algorithm; the experiments and results are presented in Section 4 followed by a related discussion; finally, the conclusions are addressed in Section 5.

## 2. System Model and Body-Shadowing Influence Analysis

This paper mainly focuses on the range-based BLE positioning, and the system model is presented in this section, together with the body-shadowing influence analysis on RSS-based ranging and positioning. It should be noted that the shadowing error in RSS measurements is named Body-Shadowing Influence Error (BSIE) in this paper.

### 2.1. System Model

**Figure 1:**
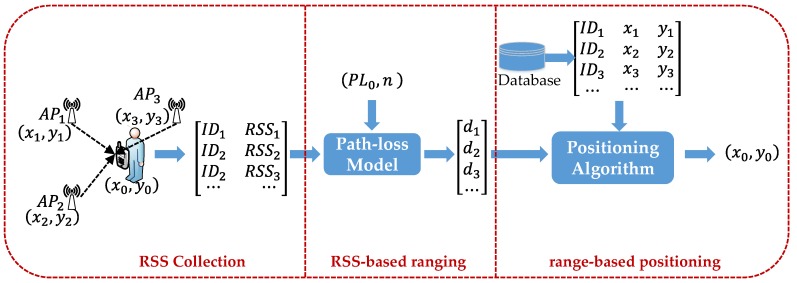
System model of range-based Bluetooth Low Energy (BLE) positioning.

The system model of the range-based indoor BLE positioning system utilized in this paper is shown in Figure 1. This positioning system consists of three main modules: RSS collection, RSS-based ranging, and range-based positioning. The RSS collection module collects the RSSs from hearable APs at a UP, and stores the RSS data for position estimation. The RSS-based ranging module calculates the distance between one AP and a UP (AP–UP distance) based on the PLM with environment-dependent parameters, and the range-based positioning module utilizes localization algorithms, like the Least-Square (LS) method and the WKNN, to obtain the final estimation of the positioning system. The BSIE in measured RSS could impair both the RSS-based ranging and the range-based positioning, as will be analyzed in detail in following sections.

where *AP_k_* (*k* = 1,2,3) is the *k*-th Bluetooth anchor point and $\left(x_{k},y_{k}\right)\;\left(k=1,2,3\right)$ is the coordinate of it. $\left(x_{0},y_{0}\right)$ is the coordinate of the unknown point (UP). *ID_k_* and *RSS_k_* are the identification and received signal strength of the *k*-th AP, respectively. *PL_0_* is the transmitting power of Bluetooth AP, and *n* is an environment-depended parameter. *d_k_* is the calculated distance between the *k*-th AP and the UP.

### 2.2. Body-Shadowing Influence on Ranging

The RSS-based ranging algorithm calculates the AP–UP distance based on PLM, which is a theoretical mathematical model representing the relationship between the transmission loss and the distance of wireless signals. The widely used PLM in the literature is shown in Equation (1).

(1) $$PL_{d}=PL_{0}+10n\log\frac{d}{d_{0}}+X_{\sigma }$$

where *PL_d_* is the path-loss in dBm of the wireless signals after transmitting to a distance of *d*, *PL*_0_ is the path-loss after transmitting to a distance of *d*_0_, *d*_0_ is the reference distance, usually set to one meter, *n* is an environment-depended parameter representing the loss coefficient, and $X_{\sigma }$ represents the shadowing error and is generally considered as a normally distributed variable.

In general, the RSS collected at the UP from one AP is the difference between the transmitted power of AP and the corresponding path-loss, as shown in Equation (2):

(2) $$RSS=P-PL_{d}$$

where *P* is the transmitted power of AP, and *PL_d_* is the path-loss. According to Equations (1) and (2), the relationship between RSS and the corresponding AP–UP distance can be derived as follows:

(3) $$RSS=P-PL_{0}-10n\log\frac{d}{d_{0}}+X_{\sigma }$$

According to Equation (3), the RSS-based ranging can be obtained, and the AP–UP distance can be calculated.

RSS-based ranging needs neither synchronization between an AP and a UP, which is necessary in Time-of-Arrival (TOA) measurement, nor Round-Trip Time (RTT) measurements to implement ranging. Meanwhile, this ranging method has low computational complexity and could achieve an accurate ranging performance when the parameters in PLM are selected or modified appropriately according to the environment. However, RSS measurements in indoor environments would have a BSIE because of the presence of human bodies. In order to make the following analysis comprehensive but not complicated, this paper makes the assumptions that the BSIE is an added noise in RSS, and the $X_{\sigma }$ without body-shadowing error is stable in the same point. Without loss of generality, the reference distance *d*_0_ is set to 1 m. The mathematical relationship between BSIE and ranging error can be derived as follows:

(4) $$RSS_{M}=RSS-BSIE=P-PL_{0}-10n\log\frac{d_{M}}{d_{0}}+X_{\sigma }$$

(5) $$BSIE=RSS-RSS_{M}=10n\log\frac{d_{M}}{d}$$

(6) $$\Delta d=d_{M}-d=d\times \left(10^{\frac{BSIE}{10n}}-1\right)$$

where *RSS_M_* is the measured RSS with BSIE, *BSIE* is the body-shadowing error in dB, $d_{M}$ is the AP–UP distance calculated on the basis of *RSS_M_*, *d* is the AP–UP distance without body-shadowing influence, and Δd stands for the ranging error caused by body-shadowing.

**Figure 2:**
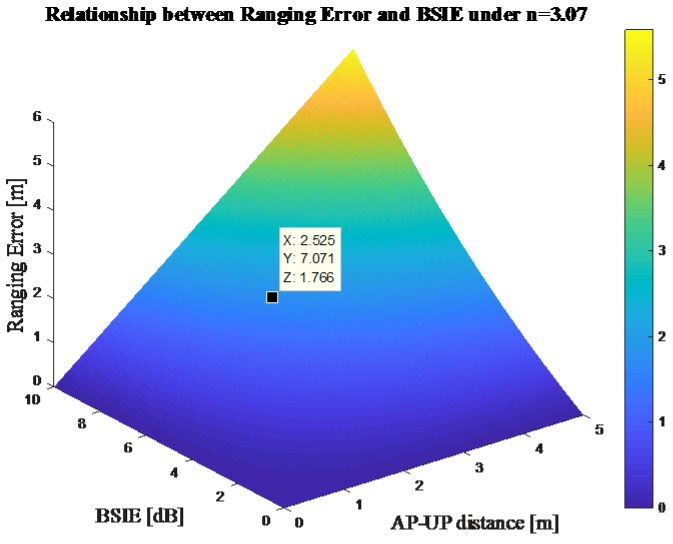
Relationship between ranging error and Body-Shadowing Influence Error (BSIE). UP: unknown point.

According to Equation (6), the ranging error is directly proportional both to the exponent of the BSIE and to the AP–UP distance. In general, the BSIE in indoor environments could be regarded as a constant about 6–8 dB [25], and the loss coefficient *n* could be set to 3.07 in indoor office environments [26]. The range of the AP–UP distance is decided by the deployment of APs and could be set within 5 m. According to these assumptions and Equation (6), the ranging error lying in body-shadowing could reach 1.8 m, which is a large bias in indoor localization. Figure 2 also shows the relationship between the ranging error and the BSIE when the loss coefficient is set to 3.07.

### 2.3. Body-Shadowing Influence on Positioning

According to the analysis in Section 2.2, the body-shadowing influence on RSS measurements could bring ranging errors, which, as a result, would cause large positioning errors. Meanwhile, the RSS attenuation caused by body-shadowing could also affect the construction of the fingerprint database and the matching in fingerprint-based positioning. This paper focuses on the analysis of range-based positioning and takes trilateration and the WKNN algorithm as examples for the following derivation.

#### 2.3.1. Influence on Positioning Using Trilateration

Positioning based on trilateration can be simplified as a problem which calculates the solution of the equations shown in Equation (7):

(7) $$\{\begin{array}{c} \sqrt{{\left(x-x_{1}\right)}^{2}+{\left(y-y_{1}\right)}^{2}+{\left(z-z_{1}\right)}^{2}}=d_{1}\\ \sqrt{{\left(x-x_{2}\right)}^{2}+{\left(y-y_{2}\right)}^{2}+{\left(z-z_{2}\right)}^{2}}=d_{2}\\ \sqrt{{\left(x-x_{3}\right)}^{2}+{\left(y-y_{3}\right)}^{2}+{\left(z-z_{3}\right)}^{2}}=d_{3}\\ \sqrt{{\left(x-x_{4}\right)}^{2}+{\left(y-y_{4}\right)}^{2}+{\left(z-z_{4}\right)}^{2}}=d_{4} \end{array}$$

where (x,y,z) represent the coordinates of UP, $\left(x_{k},y_{k},z_{k}\right),k=1,2,3,4$ are the coordinates of AP*_k_*, and $d_{k},k=1,2,3,4$ stands for the ranging results based on RSS and PLM mentioned in Section 2.2. This paper utilizes the LS method to derive the localization results based on trilateration as follows in Equations (8)–(10):

(8) $${\left(x-x_{k}\right)}^{2}+{\left(y-y_{k}\right)}^{2}+{\left(z-z_{k}\right)}^{2}-\left({\left(x-x_{1}\right)}^{2}+{\left(y-y_{1}\right)}^{2}+{\left(z-z_{1}\right)}^{2}\right)=d_{k}^{2}-d_{1}^{2}$$

(9) $$2\left(x_{k}-x_{1}\right)x+2\left(y_{k}-y_{1}\right)y+2\left(z_{k}-z_{1}\right)z=d_{1}^{2}-d_{k}^{2}+\left(x_{k}^{2}-x_{1}^{2}\right)+\left(y_{k}^{2}-y_{1}^{2}\right)+\left(z_{k}^{2}-z_{1}^{2}\right)$$

(10) $$L={\left(A^{T}A\right)}^{-1}A^{T}C$$

where the vector $L={\left(x,y,z\right)}^{T}$ represents the localization results, and the definitions of matrix *A* and *C* are shown in the following Equations (11) and (12). The parameter *k* in Equations (8) and (9) equals to 2,3,4.

(11) $$A=\left[\begin{array}{ccc} 2\left(x_{2}-x_{1}\right) & 2\left(y_{2}-y_{1}\right) & 2\left(z_{2}-z_{1}\right)\\ 2\left(x_{3}-x_{1}\right) & 2\left(y_{3}-y_{1}\right) & 2\left(z_{3}-z_{1}\right)\\ 2\left(x_{4}-x_{1}\right) & 2\left(y_{4}-y_{1}\right) & 2\left(z_{4}-z_{1}\right) \end{array}\right]$$

(12) $$C=\left[\begin{array}{c} d_{1}^{2}-d_{2}^{2}+x_{2}^{2}-x_{1}^{2}+y_{2}^{2}-y_{1}^{2}+z_{2}^{2}-z_{1}^{2}\\ d_{1}^{2}-d_{3}^{2}+x_{3}^{2}-x_{1}^{2}+y_{3}^{2}-y_{1}^{2}+z_{3}^{2}-z_{1}^{2}\\ d_{1}^{2}-d_{4}^{2}+x_{4}^{2}-x_{1}^{2}+y_{4}^{2}-y_{1}^{2}+z_{4}^{2}-z_{1}^{2} \end{array}\right]$$

According to Equations (10)–(12), *A* is a constant matrix when the deployment of APs is fixed. The localization error lies in the measurements of the AP–UP distance, and the BSIE will occur when the signal’s transmission path is blocked by human bodies. In order to evaluate the body-shadowing influence on positioning, we introduce the ranging error analyzed in Section 2.2 into Equation (12). Without loss of generality, we assume that the AP_1_ is under a body-shadowing situation, which in other words means that there is a ranging error $\Delta d_{1}$ in the measured distance *d*_1_. The positioning results under this situation can be derived as follows in Equations (13)–(15). Body-shadowing situations on other APs could be evaluated similarly to this situation.

(13) $$C_{M}=\left[\begin{array}{c} {\left(d_{1}+\Delta d_{1}\right)}^{2}-d_{2}^{2}+x_{2}^{2}-x_{1}^{2}+y_{2}^{2}-y_{1}^{2}+z_{2}^{2}-z_{1}^{2}\\ {\left(d_{1}+\Delta d_{1}\right)}^{2}-d_{3}^{2}+x_{3}^{2}-x_{1}^{2}+y_{3}^{2}-y_{1}^{2}+z_{3}^{2}-z_{1}^{2}\\ {\left(d_{1}+\Delta d_{1}\right)}^{2}-d_{4}^{2}+x_{4}^{2}-x_{1}^{2}+y_{4}^{2}-y_{1}^{2}+z_{4}^{2}-z_{1}^{2} \end{array}\right]\overset{\Delta }{=}C+\Delta C$$

(14) $$\Delta C=\left[\begin{array}{c} 2d_{1}\Delta d_{1}+\Delta d_{1}^{2}\\ 2d_{1}\Delta d_{1}+\Delta d_{1}^{2}\\ 2d_{1}\Delta d_{1}+\Delta d_{1}^{2} \end{array}\right],\;\Delta d_{1}=d_{1}\times \left(10^{\frac{BSIE_{1}}{10n}}-1\right)$$

(15) $$L_{M}={\left(A^{T}A\right)}^{-1}A^{T}C_{M}=L+{\left(A^{T}A\right)}^{-1}A^{T}\Delta C$$

where $C_{M}$ is the matrix *C* under a body-shadowing situation, ΔC is an error matrix caused by body-shadowing, $L_{M}={\left(x_{M},y_{M},z_{M}\right)}^{T}$ is the positioning result corresponding to $C_{M}$, $\Delta d_{1}$ is the ranging error in AP_1_–UP distance calculation, and *BSIE*_1_ is the corresponding body-shadowing influence error. According to Equations (10) and (15), the localization error caused by body-shadowing in range-based positioning systems can be represented as follows:

(16) $$Error=||L_{M}-L_{2}||=||{\left(A^{T}A\right)}^{-1}A^{T}\Delta C{||}_{2}$$

where *Error* stands for the localization error in meters and ${||⋇||}_{2}$ is the 2-norm operation of the matrix.

**Figure 3:**
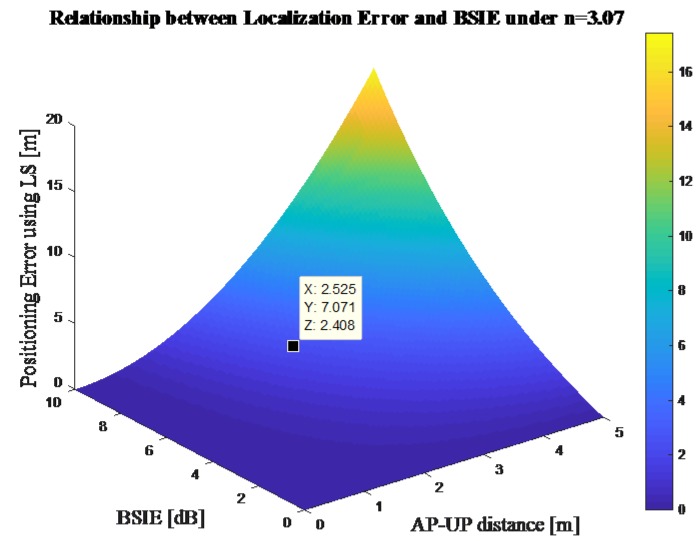
Relationship between the localization error and the BSIE using the LS method.

In order to represent the relationship between localization error and BSIE, this paper makes the assumptions that all the distances between APs are 5 m and the coefficient *n* is 3.07. According to Equation (16), the relationship between the localization error and the BSIE under the situation that only one AP is shadowed can be represented as in Figure 3.

#### 2.3.2. Influence on Positioning Using WKNN

Range-based positioning utilizing WKNN can be considered as a geometry method to estimate the position of a UP. The WKNN algorithm selects the k nearest APs of a UP based on the measured RSS, and calculates the weighted geometric center of the selected APs for localization estimation. The estimated coordinates of a UP using WKNN can be described by Equations (17)–(19). Considering that the APs are usually deployed on the ceilings, this section only evaluates the BSIE influence in the X-Y plane.

(17) $$\left(x_{0},y_{0}\right)=\left(\overset{K}{\underset{i=1}{\sum }}w_{i}\times x_{i},\overset{K}{\underset{i=1}{\sum }}w_{i}\times y_{i}\right)$$

(18) $$w_{i}=w_{i}^{'}/\overset{K}{\underset{i=1}{\sum }}w_{i}^{'}$$

(19) $$w_{i}^{'}=1/d_{i}$$

where $d_{i}$ is the calculated distance between a UP and the *i*-th selected neighboring AP, *K* is the number of selected APs for WKNN positioning, $w_{i}^{\prime }$ stands for non-normalized weight of the *i*-th neighboring AP, $w_{i}$ is the corresponding normalized weight, and $\left(x_{i},y_{i}\right)$ and $\left(x_{0},y_{0}\right)$ are the coordinates of *i*-th AP and the localization estimation using WKNN, respectively.

In order to make the following derivations simple but comprehensible, we set the parameter *K* in Equation (17) as 2, and make the assumption that the BSIE in measured RSS does not affect the AP selection results. It should be noted that in some real indoor environments, the BSIE could probability affect the AP selection in WKNN, which can bring a large positioning error for positioning using WKNN. With the assumptions above, the position estimation results can be derived as follows:

(20) $$\left(x_{0},y_{0}\right)=\left(\frac{d_{2}}{d_{1}+d_{2}}x_{1}+\frac{d_{1}}{d_{1}+d_{2}}x_{2},\frac{d_{2}}{d_{1}+d_{2}}y_{1}+\frac{d_{1}}{d_{1}+d_{2}}y_{2}\right)$$

In order to evaluate the BSIE influence on positioning using WKNN, we also introduce the ranging error analyzed in Section 2.2 into Equation (20). Without loss of generality, we assume that the measured RSS from AP_1_ exists the BSIE. Then, the estimated position of a UP under body-shadowing can be derived as follows:

(21) $$\left(x_{M},y_{M}\right)=\left(\frac{d_{2}}{d_{1}+\Delta d_{1}+d_{2}}x_{1}+\frac{d_{1}+\Delta d_{1}}{d_{1}+\Delta d_{1}+d_{2}}x_{2},\frac{d_{2}}{d_{1}+\Delta d_{1}+d_{2}}y_{1}+\frac{d_{1}+\Delta d_{1}}{d_{1}+\Delta d_{1}+d_{2}}y_{2}\right)$$

where $\left(x_{M},y_{M}\right)$ is the positioning results using WKNN under a body-shadowing situation and $\Delta d_{1}$ is the ranging error caused by BSIE. The positioning error using WKNN can be shown as follows:

(22) $$error_{w}=\sqrt{{\left(x_{M}-x_{0}\right)}^{2}+{\left(y_{M}-y_{0}\right)}^{2}}=\frac{\Delta d_{1}\times d_{2}}{\left(d_{1}+d_{2}\right)\times \left(d_{1}+\Delta d_{1}+d_{2}\right)}\times d_{12}$$

where $error_{w}$ is the positioning error using WKNN and $d_{12}$ is the distance between AP_1_ and AP_2_. Considering that the deployment of APs is usually not very high up on the ceilings, this section assumes that the approximately equals to $d_{1}+d_{2}$, and the relationship between the positioning error and the BSIE can be derived by Equation (23), according to Equations (14) and (22):

(23) $$error_{w}=\frac{d_{1}\times \left(10^{\frac{BSIE_{1}}{10n}}-1\right)\times \left(d_{12}-d_{1}\right)}{\left(d_{12}+d_{1}\times \left(10^{\frac{BSIE_{1}}{10n}}-1\right)\right)}$$

**Figure 4:**
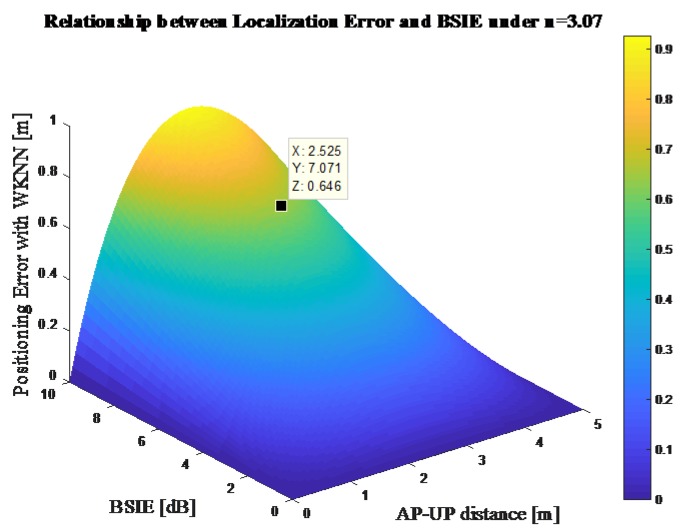
Relationship between the localization error and the BSIE using Weighted K-Nearest Neighbor (WKNN).

The relationship between the positioning error and the BSIE can also be shown as in Figure 4, with the parameters $d_{12}$ and *n* set as 5 m and 3.07, respectively.

### 2.4. Factors that May Affect Body-Shadowing Influence Error

**Figure 5:**
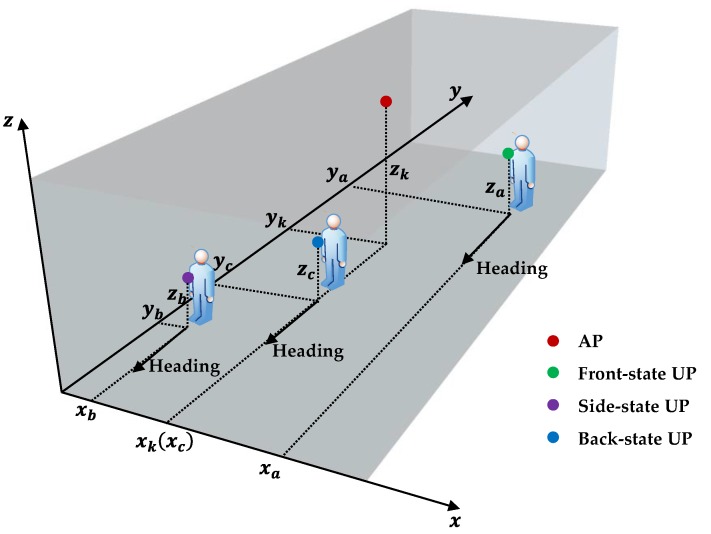
The description of the three shadowing states considered in this work.

Body-shadowing could considerable impair both ranging and positioning based on RSS, according to the analyses in Section 2.1 and Section 2.2. Meanwhile, there are still some factors that may affect the value of the BSIE when considering body-shadowing error compensation. The shadowing angle from AP to UP, the distance between shadowed AP and UP, the features of the human bodies that cause body-shadowing are some of the factors that can cause different BSIEs. The shadowing angle from AP to UP corresponds to the relative position of AP and UP in this research and can be divided into three states: back, front, and side. The distance factor indicates how far is AP from UP in the X-Y plane, given that the APs are generally deployed on the ceilings. The features of the human bodies mainly consist of the human’s gender, height, and weight. This paper mainly focuses on the shadowing angle and the distance factors in the following analyses on BSIE compensation. Moreover, the shadowing angles are decided on the basis of the relative position of AP and UP, and the descriptions of these three shadowing states, namely, front, side, and back, used in this paper are shown in Figure 5.

The “front-state” is the situation in which the AP is in front of the UP, corresponding to the green point in Figure 5. The “back-state” is the situation in which the AP is behind the UP, as indicated by the blue point. The “side-state” is the situation in which the AP is behind and on the side of the UP, as indicated by the purple point. In other words, the “side-state” means that the Bluetooth signal is partly shadowed by the human body. The range of coordinates for these three shadowing states can be represented by Equation (24) according to the description in Figure 5:

(24) $$shadowing\;state=\{\begin{array}{c} front,\;\;\;\;\;\;\;\;\;when:y_{a}\geq y_{k}\;and\;x_{a}\in R\\ back,\;\;\;\;\;\;\;\;\;when:y_{c}<y_{k}\;and\;x_{c}=x_{k}\\ side,\;\;\;\;\;\;\;\;\;when:y_{b}<y_{k}\;and\;x_{b}\neq x_{k} \end{array}$$

## 3. BLE Positioning with Body-Shadowing Error Compensation

Since body-shadowing, which could bring large localization errors, is an unavoidable problem for indoor positioning systems, an error compensation should be conducted when implementing RSS-based positioning. This section designs an IMU-aided body-shadowing detection strategy based on the coarse positioning results and establishes the BSIE compensation model based on the empirical data. The BP-BEC algorithm is then proposed to mitigate the BSIE for indoor BLE positioning.

### 3.1. Body-Shadowing Detection Strategy

In order to compensate the BSIE in the measured RSS, the APs which are shadowed by human bodies should be detected firstly. Cully et al. [20] utilized a manual differentiation of body-shadowing situations based on the time information and location information. Other statistical methods to detect body-shadowing conditions have also been shown in [27]. The detection strategies mentioned above might not be intelligent or flexible enough to detect the body-shadowing situation in real-time localization. This paper utilizes the coarse BLE positioning results and the heading information obtained from IMU to detect the body-shadowing conditions.

**Figure 6:**
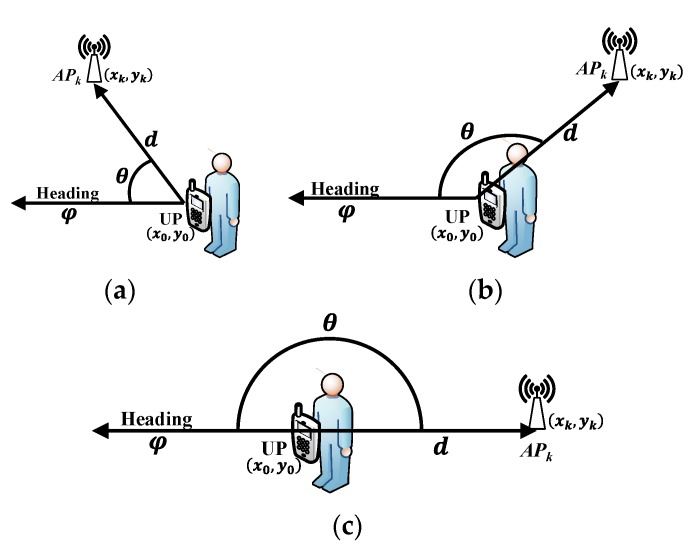
Body-shadowing detection strategy diagram: (**a**) diagram of the front condition; (**b**) diagram of the side condition; (**c**) diagram of the back condition.

IMU can measure one’s attitude and acceleration using accelerometers and gyroscopes and has been generally integrated in off-the-shelf smartphones. The heading information could be derived according to ones’ angular velocity and acceleration based on the iteration of quaternion [28]. This paper makes the assumption that the smartphone is carried in front of the human body when is located, and the heading information could be used to represent the orientation of the human body. In addition, the body-shadowing detection strategy in this paper utilizes the projections of AP and UP in the X-Y plane and the heading information to distinguish the shadowing states mentioned in Figure 5. The diagram of the proposed strategy is shown in Figure 6, and the definitions of the symbols in Figure 6 are given in Table 1.

As shown in Figure 6, the proposed body-shadowing detection strategy could detect three shadowing conditions in the shadowing angle factor mentioned in Section 2.4. This strategy utilizes the cosine of shadowing angle θ to distinguish these three states, and the mathematical description of the body-shadowing detection strategy can be represented by the following Equations (25) and (26). Given that there may be noise in measurements and calculations, the boundaries of the “front-side” state and “side-back” state are both set to a range of 15°, around 0° and 180°. As a result, the thresholds are set to −0.2588 and −0.9659 for the “front-side” and “side-back” states, respectively.

(25) $$\cos\theta =\frac{\left(x_{k}-x_{1},y_{k}-y_{1}\right)\cdot \left(\sin\varphi ,\cos\varphi \right)}{\sqrt{{\left(x_{k}-x_{1}\right)}^{2}+{\left(y_{k}-y_{1}\right)}^{2}}}=\frac{\left(x_{k}-x_{1}\right)\sin\varphi +\left(y_{k}-y_{1}\right)\cos\varphi }{\sqrt{{\left(x_{k}-x_{1}\right)}^{2}+{\left(y_{k}-y_{1}\right)}^{2}}}$$

(26) $$shadowing\_state=\{\begin{array}{c} front,\;\;\;\;\;\;\;\;\;\;\;\;\;\;\;\;\;\;\;when:\cos\theta \geq -0.2588\\ side,\;\;when:-0.9659<\cos\theta <-0.2588\\ back,\;\;\;\;\;\;\;\;\;\;\;\;\;\;\;\;\;\;\;\;when:\cos\theta \leq -0.9659 \end{array}$$

### 3.2. BSIE Compensation Model

Considering that both the shadowing angle and the distance between shadowed AP and UP can affect the value of the BSIE, the BSIE compensation model should consist of these two parameters and have the general expression shown in Equation (27):

(27) $$BSIE_{compensate}=f\left(d,\theta \right)$$

where $BSIE_{compensate}$ is the BSIE value in dB, which should be compensated in the measured RSS to obtain an accurate ranging result, and f(d,θ) is an abstract function of the shadowing angle θ and the distance d between shadowed AP and UP. 

This paper conducts experiments on the relationship among the BSIE, the shadowing angle, and the distance, and specifies the function in the BSIE compensation model on the basis of the analyses mentioned in Section 2 and the empirical data obtained. The BSIE compensation model used in this paper is represented as follows by Equation (28), and the experimental results corresponding to this model are shown in Section 4.2.

(28) $$f(\theta )=\{\begin{array}{c} \;\;\;\;\;\;\;0,\;\;\;\;\;\;\;\;\;\;\;\;\;\;\;\;\;\;\;\;\;\;\;\;\;\;\;\;\;\;\;\;\;\;\;\;\;\;\;\;\;when:\cos\theta \geq -0.2588\\ 10n\log(\frac{\sigma_{1}}{d}+\mu_{1}),\;\;when:-0.9659<\cos\theta <-0.2588\\ 10n\log(\frac{\sigma_{2}}{d}+\mu_{2}),\;\;\;\;\;\;\;\;\;\;\;\;\;\;\;\;\;\;\;\;when:\cos\theta \leq -0.9659 \end{array}$$

where $\left(\sigma_{1},\mu_{1}\right)$ and $\left(\sigma_{2},\mu_{2}\right)$ are the compensation coefficients for “side” shadowing and “back” shadowing mentioned in Section 3.1, respectively. The values of these compensation coefficients are given in Section 4.2 according to curve fitting of the empirical data.

### 3.3. BP-BEC Algorithm

**Figure 7:**
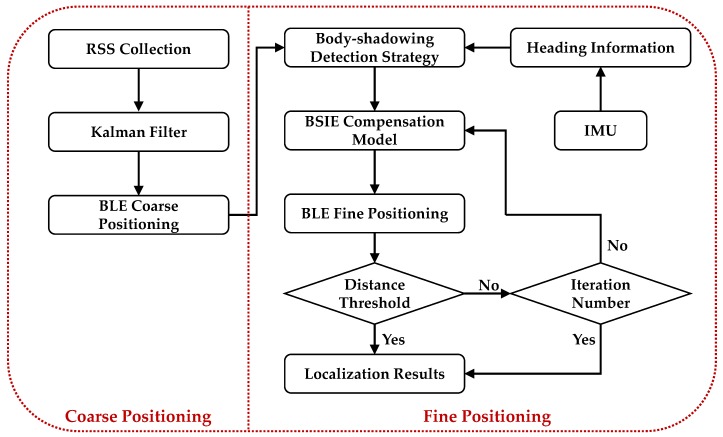
The flow chart of the body-shadowing error compensation (BP-BEC) algorithm.

Based on the body-shadowing detection strategy and the BSIE compensation model, the BP-BEC algorithm is proposed to mitigate the effect of body-shadowing and improve the performance of positioning systems in terms of accuracy and robustness. The flow chart of the BP-BEC algorithm is shown in Figure 7. The BP-BEC algorithm performs positioning process in two stages: coarse positioning and fine positioning. In the coarse-positioning stage, the RSS measurements from different APs are collected at a UP, and a Kalman filter is utilized to filter the measurement noise and to smooth the measurements. Then, the LS method or WKNN algorithm is implemented to obtain the coarse positioning results. It should be noted that these positioning results are called coarse results because of the existence of the body-shadowing error.

In the fine-positioning stage of the BP-BEC algorithm, the heading information from IMU and the coarse-positioning results are integrated to distinguish the shadowed APs from all hearable APs on the basis of the detection strategy proposed in Section 3.1. Then, the BSIE compensation model in Section 3.2 is applied to compensate the measured RSSs of shadowed APs. After the error compensation process, the positioning process is conducted again using the compensated RSS, and a new location result is obtained. The processes of compensation and positioning are iterated until the distance between two positioning results is smaller than the given threshold, or the iteration number is larger than the given number. A fine-positioning result could be obtained after the iteration, and this is the final output result of the BP-BEC. In this paper, the distance threshold is set as 0.1 m through measurements and empirical data, and the iteration number is set to 50 with the consideration of real-time localization.

## 4. Experiments and Results

### 4.1 Experimental Scenario and Implementation

**Figure 8:**
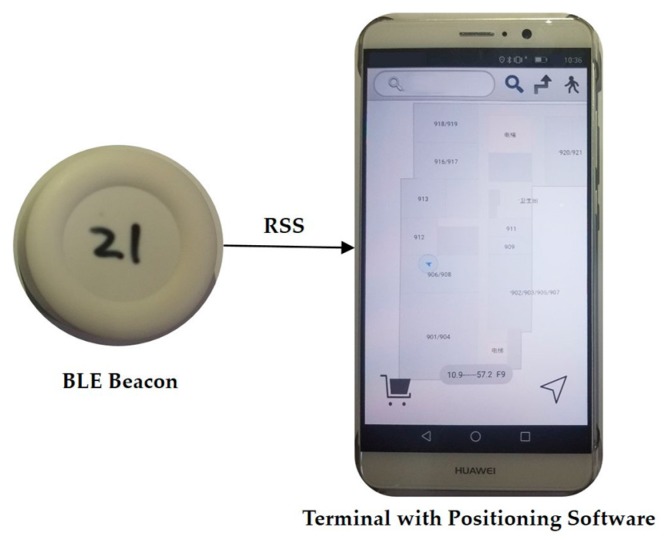
The BLE beacons and terminal used in this work.

We conducted our experiments in two indoor body-shadowing environments in order to evaluate the body-shadowing effect and the performance of the proposed BP-BEC algorithm. One of the experimental environments consisted of two indoor rooms in our office (indicated as Test bed 1), and the other was a space at the ninth floor of the Scientific Research Building in our university (indicated as Test bed 2). All the RSSs were collected using a smartphone with Bluetooth module (named terminal), which was held in front of the body by the testers. The BLE beacons and the terminal used in this work are shown in Figure 8.

**Figure 9:**
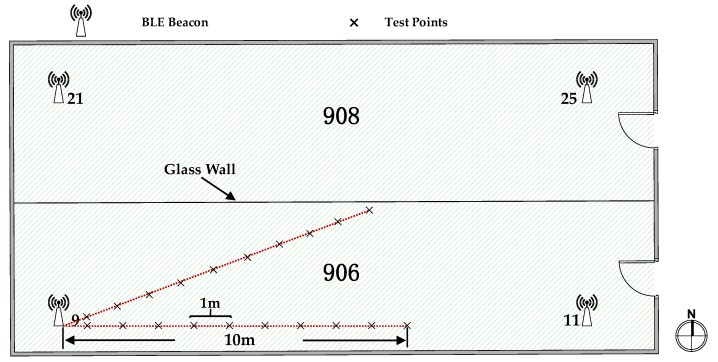
Experimental environment for Test bed 1.

Figure 9 shows the planer graph and the deployment of the BLE beacons (also named APs) in the Test bed 1. This test bed, with an area of 8 m by 16.46 m, consists of two office rooms, which are separated by a glass wall in the middle. The whole area in Test bed 1 was covered with Bluetooth signals from four beacons with different Universally Unique Identifiers (UUIDs), and the beacons were deployed around the room corners up on the ceilings, as shown in Figure 9. The test points are indicated by black crosses. The operating frequency of the BLE beacons was 2.4 GHz, and the signal transmitting frequency was about 2 Hz, which, in other words, means that we could collect two RSSs in one second. We conducted experiments to evaluate the body-shadowing influence on RSS in Test bed 1.

**Figure 10:**
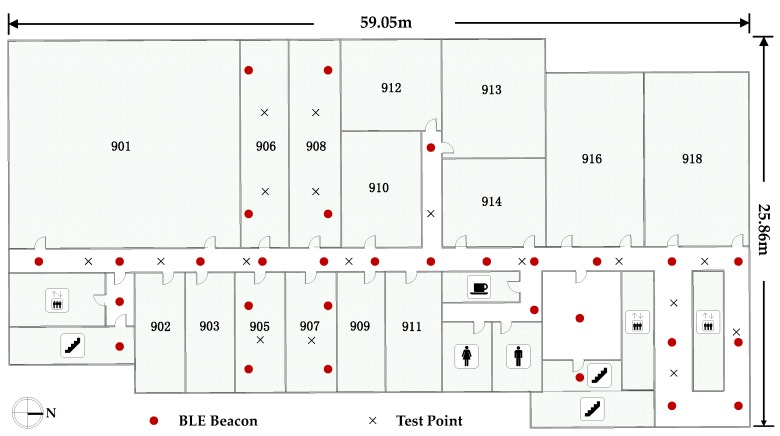
Experimental environment for Test bed 2.

The planer graph of Test bed 2 and the deployment of APs are shown in Figure 10, along with the implementation of the test points. This area is 25.86 m wide and 59.05 m long, and was covered with Bluetooth signals from 30 BLE beacons with unique UUIDs. The deployment of the beacons was ordered as T-shape lines with an interval of about 5 m, indicated by the red dots in Figure 10. Experiments on positioning performance evaluation were conducted in Test bed 2, and the locations of the test points are shown as black crosses in Figure 10.

### 4.2. BSIE Evaluation and Analysis

Experiments were conducted in Test bed 1 to evaluate the influence of body-shadowing on RSS and the factors that affect the body-shadowing error. We measured RSSs from one AP with an AP–UP distance ranging from 1m to 10m and with different shadowing angles, namely, “front”, “side”, and “back”. We collected 240 pieces of RSS data at each AP–UP distance with each shadowing angle for one pair of AP­–UP, and four pairs of AP–UP (AP9–UP, AP11–UP, AP21–UP, and AP25–UP) were evaluated, so that, totally, 28,800 pieces of RSS data were collected for BSIE evaluation. Without loss of generality, this section chooses the AP9–UP pair to verify and to represent the body-shadowing influence on RSS. The mean values of BSIE at the same test point for the same shadowing angle of all the test points were calculated for the analyses. This paper considered the RSS of the “front” state as a reference; the measured BSIE can be derived as follows by Equations (29) and (30).

(29) $$BSIE_{side,d}=\overset{4}{\underset{k=1}{\sum }}\left(RSS_{front,d,k}-RSS_{side,d,k}\right)$$

(30) $$BSIE_{back,d}=\overset{4}{\underset{k=1}{\sum }}\left(RSS_{front,d,k}-RSS_{back,d,k}\right)$$

where $BSIE_{side,d}$ and $BSIE_{back,d}$ are the BSIE in the “side” shadowing angle and the “back” shadowing angle at the distance d, respectively, and $RSS_{front,d,k}$, $RSS_{side,d,k}$, and $RSS_{back,d,k}$ are the mean values of filtered RSS data in these three shadowing angles at the distance d for the *k*-th AP-UP pair.

**Figure 11:**
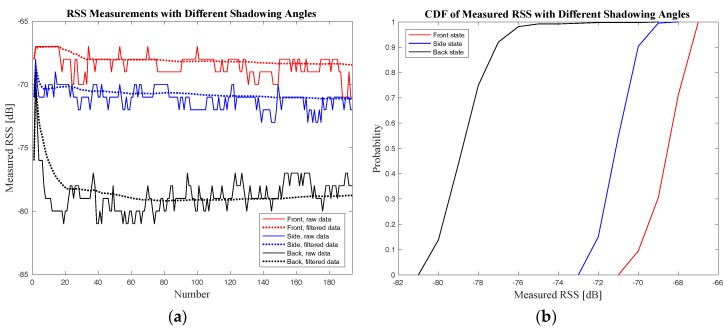
The RSS data for AP9–UP pair at a distance of 3 m for three shadowing angles: (**a**) the original measured RSS data and the filtered data through Kalman filter for the three angle states; (**b**) the Cumulative Distribution Function (CDF) of the measured RSS data for the three angle states.

The original RSS data and the filtered RSS data at a distance of 3 m for three shadowing angles of AP9–UP pair are shown in Figure 11a. The Cumulative Distribution Function (CDF) of the measured RSS is also shown in Figure 11b in order to give a better demonstration of the body-shadowing influence.

As shown in Figure 11, the body-shadowing influence on RSS is obvious and could cause a considerable attenuation in the RSS. The RSS attenuation for the AP9–UP pair under body-shadowing is 10 dB for the “back” state and 3dB for the “side” state. It can also be concluded that different shadowing angles can influence differently the RSS measurements.

**Figure 12:**
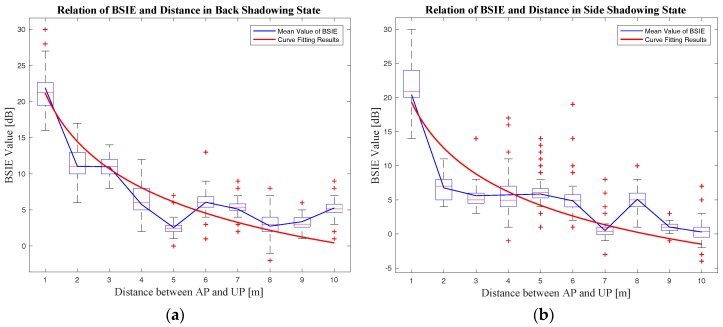
The relation between the BSIE and the AP–UP distance for different shadowing angles: (**a**) the relation between the BSIE and the distance in the “back” state and the curve fitting results; (**b**) the relation between the BSIE and the distance in the “side” state and the curve fitting results.

**Figure 13:**
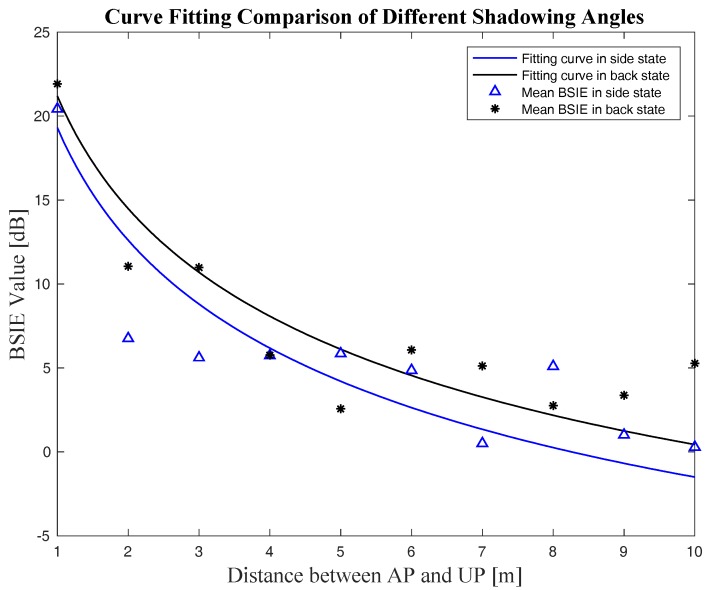
The curve fitting results comparison of different shadowing angles.

The relations between the measured BSIE and the AP–UP distance for both the “back” and the “side” shadowing angles were evaluated and represented in Figure 12. Meanwhile, the curve fitting was done based on the BSIE compensation model proposed in Section 3.2 utilizing the measured data. The fitting results are also shown in Figure 12, and the comparison of fitting curves for different shadowing angles together with the curve parameters are shown in Figure 13 and Table 2. Curve fitting was also done to estimate the parameters of PLM in our experimental environments; the *PL*_0_ was −50.06 and *n* was 2.33.

### 4.3. Positioning Performance Evaluation and Analysis

In order to evaluate the positioning performance of the BP-BEC algorithm proposed in this paper, experiments were conducted in Test bed 2, and both the positioning accuracy and the robustness of the positioning system were presented. Two evaluation indicators, namely, positioning error and robustness error, were utilized in this paper to represent the system’s performance in terms of positioning accuracy and positioning robustness, respectively. This section firstly gives the descriptions of the evaluation indicators, and then the experimental results in a static scenario are shown and discussed. Moreover, the convergence time of the BP-BEC algorithm was evaluated, and continuous positioning results using the proposed algorithm are also presented.

#### 4.3.1. Performance Evaluation Indicators

The performance evaluation indicators in this paper consist of the positioning error and the robustness error. The positioning error refers to the relative position of the real position and calculated position using the positioning algorithms of a UP, which in other words means the distance between the real position and the calculated position. The mathematical expression of the positioning error is as follows:

(31) $$error_{a,m}=\sqrt{{\left(x_{m}-x_{m}^{'}\right)}^{2}+{\left(y_{m}-y_{m}^{'}\right)}^{2}}$$

where $error_{a,m}$ stands for the positioning error at the *m*-th test point, $\left(x_{m},y_{m}\right)$ is the true coordinates of the *m*-th test point, and $\left(x_{m}^{'},y_{m}^{'}\right)$ is the localization result of the *m*-th test point.

The robustness error used in this paper represents the stability performance of the system when the body-shadowing state at a UP changes, for example from “front” to “back”. The works in [16] demonstrated that different human body’s directions can cause different attenuations in the RSS of WLAN signals, which has also been shown in Section 4.2 for Bluetooth signals. According to the characteristics of Test bed 2 in Figure 10, we selected two directions, i.e., North-facing and South-facing, in each test point for robustness evaluation. The robustness error in this paper is the difference of the positioning results under different facing directions; the corresponding mathematical expression is shown in Equation (32):

(32) $$error_{r,m}=\sqrt{{\left(x_{m,N}^{'}-x_{m,S}^{'}\right)}^{2}+{\left(y_{m,N}^{'}-y_{m,S}^{'}\right)}^{2}}$$

where $error_{r,m}$ stands for the robustness error at the *m*-th test point, and $\left(x_{m,N}^{'},y_{m,N}^{'}\right)$ and $\left(x_{m,S}^{'},y_{m,S}^{'}\right)$ are the localization results when the human with a smartphone is facing North and South, respectively.

Considering the deployment of APs and the Geometric Dilution of Precision (GDOP) when performing positioning algorithms, this paper used both the LS method and the WKNN algorithm in the BP-BEC algorithm. The LS method was applied in four rooms with good GDOP (Room 905, Room 906, Room 907, and Room 908), and the WKNN algorithm was applied in the corridor of Test bed 2. As a control, algorithms without body-shadowing error compensation (called no-BEC) were also be implemented in Test bed 2. This paper selected 17 test points, and both North-facing and South-facing RSS were collected for robustness evaluation. We collected 60 pieces of RSS data that were averaged at each point for each facing direction, providing 34 results for accuracy evaluation and 17 results for robustness evaluation.

#### 4.3.2. Positioning Accuracy Measurements and Analysis

**Figure 14:**
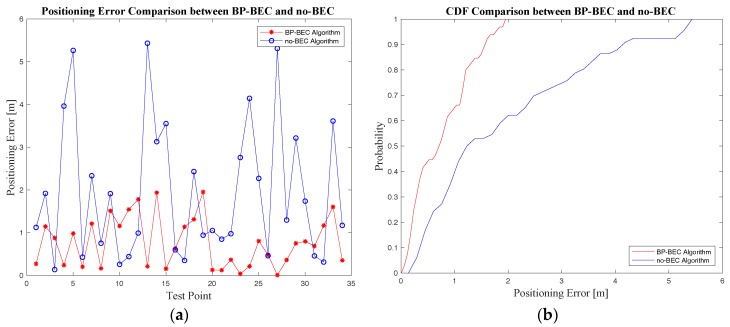
The comparison of positioning accuracy between the BP-BEC algorithm and the algorithm without body-shadowing error compensation (no-BEC): (**a**) positioning error comparison in scatter diagram; (**b**) positioning error comparison in CDF.

Experiments on positioning accuracy for both the BP-BEC algorithm and the no-BEC algorithm were designed and conducted in Test bed 2, and all the positioning errors at the test points were calculated according to Equation (30). The comparisons of positioning are shown in Figure 14 and Table 3. It can be concluded that the BP-BEC algorithm outperforms the no-BEC algorithm in positioning accuracy because of the compensation of the body-shadowing error. The mean positioning accuracy was improved by about 60.1% using BP-BEC compared to no-BEC.

#### 4.3.3. Positioning Robustness Evaluation and Analysis

**Figure 15:**
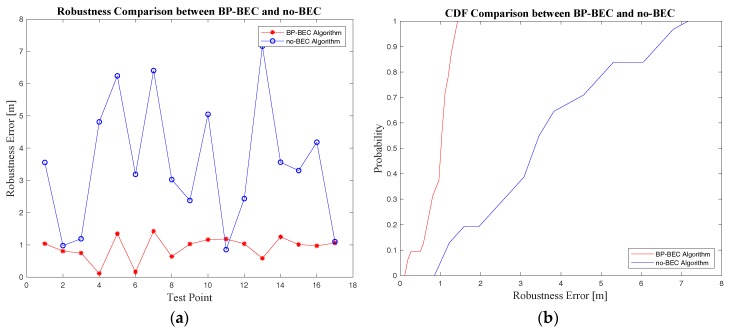
The comparison of positioning robustness between the BP-BEC algorithm and the traditional algorithm: (**a**) positioning robustness error comparison in scatter diagram; (**b**) positioning robustness error comparison in CDF.

The robustness error for all test points was calculated according to Equation (31), and the comparison in terms of positioning robustness for these two algorithms are presented in Figure 15 and Table 4. It can be concluded that the BP-BEC algorithm could improve the positioning robustness by about 73.6% compared to the no-BEC algorithm, and the mean error with different heading directions in the BP-BEC algorithm was 0.92m, compared to 3.49m in the no-BEC algorithm.

#### 4.3.4. Positioning Evaluation under a Dynamic Situation

Positioning algorithms should meet the demands of localization in dynamic environments, which, in other words, means that the speed of providing positioning results should also be considered when evaluating a positioning system. In this section, the speed of convergence for the BP-BEC algorithm and the time of providing results for the no-BEC algorithm are shown and discussed. Moreover, continuous localization trajectories in Test bed 2 for the BP-BEC and the no-BEC algorithms are also shown and analyzed.

**Figure 16:**
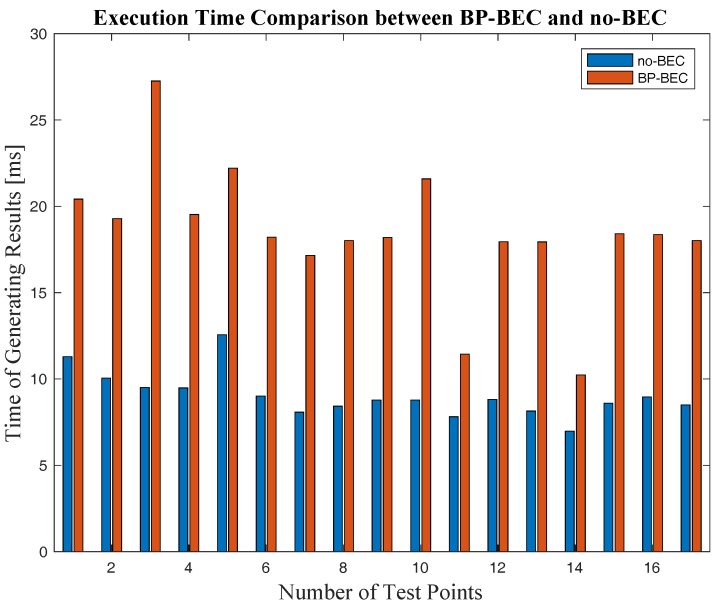
The execution time comparison between the BP-BEC and no-BEC algorithms.

For the purpose of convergence speed evaluation, both the BP-BEC and the no-BEC algorithms were used in the same computer on the collected RSS data for all the 17 test points. The execution times of these two algorithms for each test point were recorded and are shown in Figure 16.

As shown in Figure 16, the mean execution time of the BP-BEC algorithm was 18.5 ms, whereas that of the no-BEC algorithm was 9.1 ms. It can be concluded that the execution time of the BP-BEC algorithm is longer than that of the no-BEC algorithm, because there are iterative processes in the BP-BEC in order to compensate the body-shadowing error. However, the execution time of the BP-BEC algorithm is still at a millisecond level, while the walking speed of humans is usually about 0.8~1.3 m/s [29], and an amount of some tens of milliseconds is much lower than the reaction time of the human body, which means that the convergence speed would not affect the localization of a real-case walking situation.

**Figure 17:**
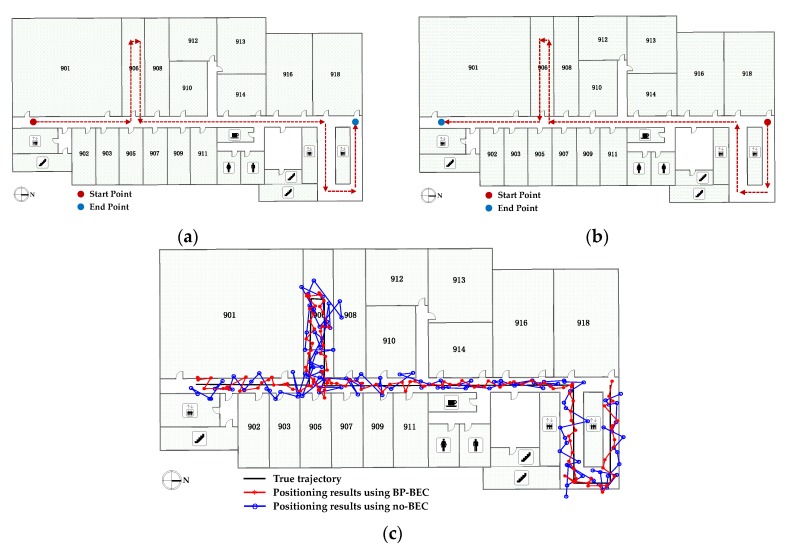

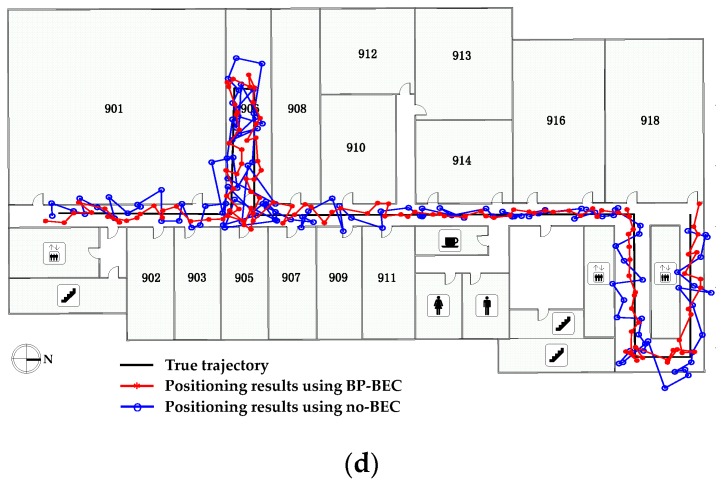
The real trajectories in Test bed 2 and the positioning results comparison between the BP-BEC and the no-BECalgorithms: (**a**) the real trajectory 1 in Test bed 2; (**b**) the real trajectory 2 in Test bed 2; (**c**) the positioning results comparison along trajectory 1; (**d**) the positioning results comparison along trajectory 2.

In order to evaluate the performance of the proposed algorithm under a dynamic situation, experiments were conducted in Test bed 2, and two trajectories were selected. The device was held by the tester in front of the body while the tester moved at a walking speed. The real trajectories in Test bed 2 are shown in Figure 17a,b. With an IMU-aided method, the RSS data at a UP was collected and filtered in each step interval for both the BP-BEC and the no-BEC algorithms to generate positioning results, and the positioning results in a moving condition along the trajectories for both algorithms are shown in Figure 17c,d. It can be concluded that the real-case situation localization is not affected by the convergence speed of the BP-BEC algorithm, and both the positioning accuracy and the robustness were improved compared to the no-BEC algorithm.

### 4.4. Discussion of the Strengths and Weaknesses of the Proposed Method

The strength of the BP-BEC algorithm mainly consist of the improvements in both positioning accuracy and robustness, which were demonstrated by the experimental results. The BP-BEC improves the positioning accuracy and robustness by about 60.1% and 73.6%, respectively, compared to the no-BEC. In addition, our works could also provide an alternative strategy when considering the body-shadowing error mitigation. The weakness of the proposed algorithm lies in the execution time of BP-BEC algorithm. The execution time of the BP-BEC algorithm is longer than that of the no-BEC algorithm because it implies iterations in order to compensate the BSIE. However, the speed of generating localization results of the BP-BEC algorithm is still at the millisecond level, which would have an insignificant effect on real-time localizations according to the experimental results in Section 4.3.4.

## 5. Conclusions

In this paper, an IMU-aided body-shadowing error compensation method has been proposed, analyzed, and evaluated. The body-shadowing impairment on both RSS-based ranging and trilateration positioning was studied, and a mathematical expression of the relation between the BSIE and the positioning error was derived based on PLM. Factors which may affect the BSIE were given and studied, and an IMU-aided body-shadowing detection strategy was designed to distinguish the shadowed APs in the positioning system. In addition, a body-shadowing error compensation model was established and the BP-BEC algorithm was proposed to improve the positioning accuracy and robustness in indoor BLE positioning. Experiments were designed and conducted in two test beds to validate the improvement of the proposed algorithm. The results showed that the BP-BEC algorithm could improve the positioning accuracy and robustness by about 60.1% and 73.6%, respectively, compared to the no-BEC algorithm. Moreover, the speed of generating the positioning results for the BP-BEC algorithm was also evaluated and discussed. Although the convergence speed of positioning is partly sacrificed in the BP-BEC algorithm because of the body-shadowing error compensation, the localization results in a real-case situation with movements at a walking speed is not affected. Our work could be helpful for the implementation of indoor RSS-based positioning systems and it could also be a guidance for body-shadowing error mitigation and performance improvement of indoor positioning systems.

## Figures and Tables

**Table 1:** Symbol definitions in the diagram of the body-shadowing detection strategy.

Symbol	Definition
$AP_{k}$	The projection of the *k*-th anchor point, represents the shadowed Bluetooth beacon
UP	The projection of the unknown point, represents the point being located
$\left(x_{k},y_{k}\right)$	The coordinates of the *k*-th AP which is shadowed by a human body
$\left(x_{0},y_{0}\right)$	The coordinates of coarse positioning resulting from BLE positioning
d	The distance between the projections of shadowed AP and UP
φ	The heading information obtained from IMU, indicating the clockwise angle between the North and the body’s orientation, ranging from 0° to 360°
θ	The angle information utilized to detect the shadowing state, ranging from 0° to 180°

**Table 2:** The parameters for the BSIE compensation model from curve fitting.

Shadowing Angle	Parameter	Value
Side	$\mu_{1}$	6.531
	$\sigma_{1}$	0.2093
Back	$\mu_{2}$	7.854
	$\sigma_{2}$	0.2589

**Table 3:** Positioning error comparisons between the BP-BEC and the no-BEC algorithms

Algorithm	Mean Positioning Error (m)	90% Positioning Error (m)
BP-BEC	0.77	1.553
no-BEC	1.93	4.187

**Table 4:** Robustness error comparison between the BP-BEC and the no-BEC algorithms

Algorithm	Mean Robustness Error (m)	90% Robustness Error (m)
BP-BEC	0.92	1.273
no-BEC	3.49	6.42
